# Notes and News

**Published:** 1903-03-28

**Authors:** 


### Notes and News.

HOSPITAL MEETINGS AND FESTIVALS.

Hospital and Home for Incurable Children.—Annual Meeting, Saturday, March 28, at 2 Maida Vale, at 4 p.m.

St. Peter's Hospital.—Annual Meeting, Tuesday, March 31, at 5 p.m.

Royal Dental Hospital.—Annual Meeting, Tuesday, March 31, at 5.30 p.m.

West London Hospital.—Annual Meeting, Thursday, April 2, at 4 p.m.

Scarlet fever, traceable to contaminated milk, is now dying out in Edinburgh.

The Lord Mayor will preside at a meeting in aid of the Hospital for Women, Soho Square, W., at the Mansion House on Friday, March 27th, at 3 p.m. H.R.H. the Princess Christian has signified her intention of being present.

The Briton has long regarded the "grandmotherly" legislation of the United States with some contempt, but somewhat late in the day the tendency of English legislature is showing signs of following suit. The citizen, especially in the matter of health, is to be protected against himself. First we have the habitual drunkard under supervision, and now within a very short period a crusade will probably be waged against the youthful smoker. A few generations back and parents and guardians would have resented the slight thus cast upon their authority; but of late years an unfortunate contempt for authority of any description by the young makes it not an unusual occurrence that a parent applies to the police courts for assistance in the control of his child; and further, even, we have a recent example of the boy who had his father up for assault. Smoking is regarded as an essential to dignity by almost every small boy in the streets, and the police will find they have plenty to do if the Bill to prevent smoking amongst the populace under sixteen years of age passes.

An International Congress on Tuberculosis is to be held at St. Louis, United States, in July 1904.

King Edward's Hospital Fund has received £2,000, and the Brompton Hospital for Consumption and Great Ormond Street Hospital for Children £1,000 each, through the will of the late Mr. Mortimer Harris, of Brixton.

A Conference has been held at Owens College, Manchester, by the representatives of the Colleges of Victoria University on the matter of the proposed independent universities. The Privy Council has proposed joint action in some directions, and at the Conference it was pointed out that Manchester and Liverpool could settle their future course of action without waiting for Yorkshire, which has not settled the question of its representative university.

Dora, Lady Chesterfield, writes the following letter in connection with the League of Mercy:—"The people who came to hear Sir Henry Burdett at Kensington Town Hall on Thursday were so numerous that some hundreds could not gain admission. As lady president for Kensington I greatly regret any disappointment thus caused; but I rejoice to know that the interest excited in the hospitals by Sir Henry's addresses has been so great that hundreds have been turned away from several other meetings. In these circumstances may I make a practical suggestion? It is that an arrangement should be made to hold a mass meeting in the Albert Hall before Easter. All the vice-presidents, members, and associates could be invited, with those Londoners who are desirous to hear Sir Henry's address and to see his graphic pictures from the hospitals. I am assured that a large number of those who have already been present would welcome such an opportunity of coming again. We workers for the League would thus be able to meet all together, which would inspirit us greatly, and so the good work done by these meetings during the winter would be cemented and secured. If the Grand President could preside it is possible that even the Albert Hall may not be large enough to meet the demand for seats."

